# Improving access to effective care for people with chronic respiratory symptoms in low and middle income countries

**DOI:** 10.1186/1753-6561-9-S10-S3

**Published:** 2015-12-18

**Authors:** Kevin Mortimer, Luis Cuevas, Bertie Squire, Rachael Thomson, Rachel Tolhurst

**Affiliations:** 1 Liverpool School of Tropical Medicine, UK

## Abstract

Chronic respiratory symptoms are amongst the most common complaints among low and middle-income country (LMICs) populations and they are expected to remain common over the 10 to 20 year horizon. The underlying diseases (predominantly chronic obstructive pulmonary disease, asthma and tuberculosis) cause, and threaten to increasingly cause, substantial morbidity and mortality. Effective treatment is available for these conditions but LMICs health systems are not well set up to provide accessible clinical diagnostic pathways that lead to sustainable and affordable management plans especially for the chronic non communicable respiratory diseases. There is a need for clinical and academic capacity building together with well-conducted health systems research to underpin health service strengthening, policy and decision-making. There is an opportunity to integrate solutions for improving access to effective care for people with chronic respiratory symptoms with approaches to tackle other major population health issues that depend on well-functioning health services such as chronic communicable (e.g. HIV) and non-communicable (e.g. cardiovascular and metabolic) diseases.

## Introduction

Chronic respiratory diseases are common causes of morbidity among poor populations across the world, with breathlessness, chest tightness, coughing and wheezing being the main symptoms. The non-communicable airways diseases chronic obstructive pulmonary disease (COPD), asthma and - to a lesser extent - bronchiectasis dominate the picture. However other conditions including malignancies, such as bronchogenic carcinoma, Kaposi's sarcoma and mesothelioma, can cause chronic respiratory symptoms and their burden is poorly defined in most LMICs. Chronic respiratory symptoms can also be caused by infections, e.g. tuberculosis (TB), and their clinical presentation overlaps with non-communicable airways diseases. This overlap is especially important in LMICs where tuberculosis is common, as the diagnosis and management differ, and patients with chronic respiratory diseases are often managed with an incomplete diagnostic pathway.

This discussion paper will focus on the non-communicable chronic airways diseases and their interface with TB in LMICs. These conditions represent a major burden of disease and their public health importance is expected to increase in the next two decades. Although focused on chronic respiratory diseases, the health systems considerations presented here could apply to other chronic non-communicable diseases and the potential to identify health system synergies for their combined management needs to be considered.

## The burden of airways diseases

COPD and asthma are the two of the most common chronic diseases worldwide, with 80 million people having COPD and 235 million estimated to have asthma worldwide[1]. In 2005 three million people died from COPD alone, making this condition the fifth leading cause of adult death. The burden of these diseases is also increasing, and COPD is expected to be the third leading cause of death by 2030[2].

In addition to causing a high death burden, COPD and asthma result in significant morbidity, with persistent symptoms, reduced lung function and intermittent exacerbations adversely affecting functional status and quality of life.

The distribution of COPD varies around the world and factors such as the prevalence of smoking and population age distribution are associated with its prevalence. For example, Cape Town has a particularly high prevalence of COPD, very high smoking rates, high levels of occupational exposures and TB[3].

The increasingly high rates of smoking in some LMICs like China and South East Asia, together with increasing drivers of COPD like air pollution, biomass fuel use, crowding and ageing populations will make COPD a major health issue in the next decades. Smoking rates are expanding in most LMICs, even in areas with relatively low prevalence – particularly in Sub Saharan Africa – and the world faces an ongoing high risk from the smoking epidemic.

The distribution of asthma also varies worldwide, from 0.8% in Tibet and 2.4% in India to 32.6% amongst adolescents in New Zealand and 37.6% in Costa Rica. These variations can also be observed between urban and rural areas for reasons that are not well understood and may reflect differences in early life exposure to infections, allergens and air pollution. Although asthma is more common in affluent settings, symptoms are usually more severe in less affluent settings[4].

Bronchiectasis is less common than COPD and asthma, but causes chronic morbidity with intermittent exacerbations associated with infections. The global burden of this syndrome is poorly documented due to the limited access to diagnostic radiology, including High Resolution Computed Tomography (HRCT) in LMIC populations. HRCT has become a standard of care in the evaluation of people with bronchiectasis in well-resourced healthcare settings, this is likely to be some decades away in poor resourced settings where other priorities are likely to come first.

The high burden of ill health caused by chronic respiratory diseases in LMICs is accompanied by a high burden of tuberculosis too. It is also estimated that in 2012 there were 8.6 million new TB cases with 0.94 million deaths around the world[5]. TB control is the focus of major international and national control programmes and consequently receives substantially more funding in LMICs than all other chronic respiratory diseases combined. National TB programmes are universally available in LMIC, are organized as vertical programmes and receive funding from National budget allocations and international funders. The TB diagnostic pathway mostly screens patients using smear microscopy and this is often the only diagnostic approach available for patients with chronic respiratory symptoms. Although current diagnostics confirm the presence of TB in about 10-20% of patients screened, the 80-90% of patients with negative tests are left without a clear diagnosis. This group of patients is often repeatedly screened with further TB tests, empirically diagnosed as having TB and therefore inadequately treated [CAHRD Paper LH Costs]. There is thus a substantial number of individuals who receive the wrong diagnosis; and there is no clear pathway for the management of patients who are diagnosed as not having TB.

## Obstacles to accessing effective care

Many factors impede people with chronic respiratory symptoms gaining access to effective care. These could be considered at the individual, societal and health system levels and are better documented for TB than for other CRDs:

### Individual factors

A major obstacle to accessing effective care is the lack of awareness of the disease. For example, smokers often expect to have chronic respiratory symptoms and believe having these symptoms is ‘normal’. There is a wide range of population concepts of illness, including local ethno-medical models that may not easily accommodate concepts of chronicity and ‘incurability’[6]. This is often followed by a wide variety of ways in which different cultures organize themselves toward medical treatment.

To attend a health service, an individual needs to recognize the symptoms are abnormal, that symptoms are sufficiently severe to warrant attention and to perceive the gains of seeking help are greater than the costs involved. Decision-making processes are influenced by many factors, including gender, age, disability, socioeconomic status and others. For example, elderly individuals with chronic respiratory symptoms may adjust their lifestyle and attribute their symptoms to ‘age’, considering that nothing can be done or that the symptoms are not worth treating. Value judgments can be gender-aligned e.g. young married women or disabled people may feel discouraged to place value on their own health[7] or access services; masculinities in some contexts can also mitigate against seeking healthcare[8,9]. Even where care is sought and a diagnosis is achieved, chronic illness can continue to cause disruption to physical capabilities, social identity and life trajectory, all of which pose challenges for long-term management of illness and require a holistic approach to providing care[10]. For example a study of perceptions and experiences of adults and children with asthma in rural South Africa found significant psychological impacts including high levels of fear and embarrassment[11].

### Societal factors

Chronic respiratory symptoms - especially chronic productive cough - is often associated with stigma and assumed to be TB. TB has a high level of stigma that differentially affects women and men in different contexts depending on factors such as whether they are married or not[12,13]. There are also high levels of stigma towards people with asthma who are often reluctant to accept their diagnosis. In Sudan, patients prefer to regard their condition as an allergy, with a high use of treatment inhalers and frequent admission to emergency rooms coupled with a very low use of preventer treatment. There is a common belief that the use of preventers leads to drug dependency and this impacts on marriage prospects and family reputation[14].

In settings with high HIV prevalence, a diagnosis of TB is often associated with HIV adding another layer of stigma[15-17]. In settings where tobacco smoking is common there can be a society ‘lack of sympathy’ for individuals with chronic respiratory symptoms who have ‘brought these on themselves’. These societal attitudes may discourage health-seeking behaviour. Furthermore social support has been identified as important to the effective management of chronic illness but the demands on carers and increased dependence by individuals experiencing chronic disease may place strains on family relations, jeopardizing their support[10,18].

### Health system factors

**Figure 1:**
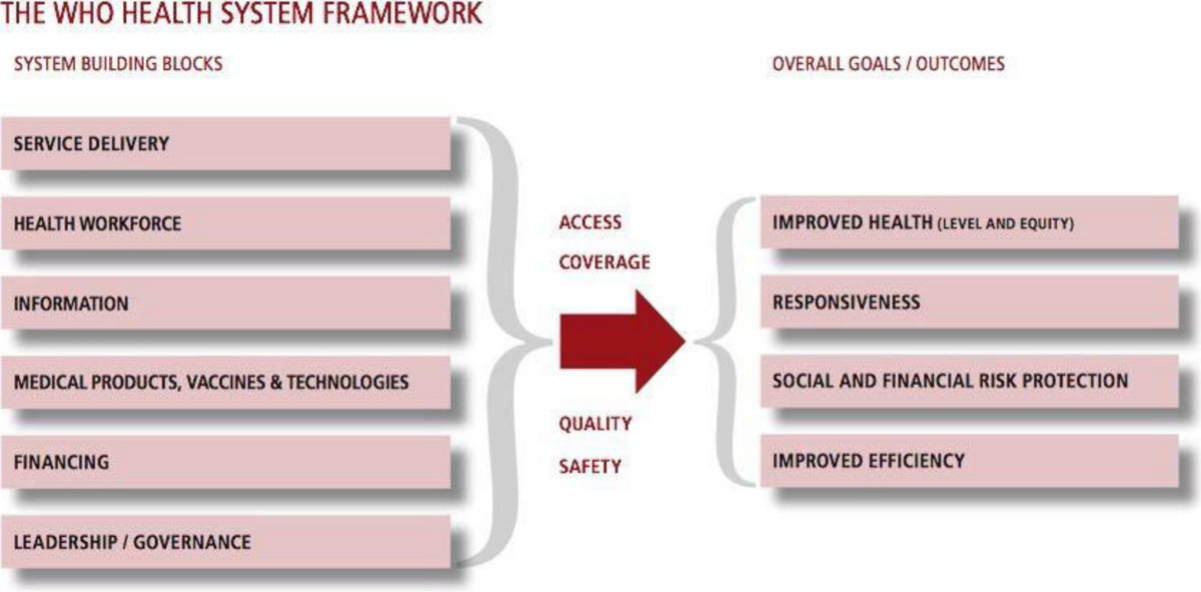
The WHO Health System Framework

To provide effective care for patients with chronic respiratory symptoms a health system needs to be in place that is 1) accessible for people in need in an equitable way, 2) adequately staffed by skilled healthcare workers, 3) responsive to health systems information, 4) adequately resourced in terms of medical products and treatments for the delivery of chronic disease and exacerbation management, 5) affordable and 6) led and governed strategically. Figure 1

### Accessibility (geographic, direct and indirect costs and social)

The individuals most in need of healthcare for chronic respiratory disease are often those who are disadvantaged on other fronts (e.g. poverty, disability, other forms of marginalization including living in remote areas), often in a way that is related to the underlying disease. For example, a woman who develops COPD as a result of her lifetime exposure to biomass smoke, who is responsible for attending to crops and looking after children, may face difficulties accessing care because of her specific roles in the family, a lack of decision making power and status and a lack of access to or control over funds to travel to seek care. A young man disabled by severe breathlessness, wheezing and coughing may face a different set of disability-driven challenges including limited educational and employment opportunities. Looking over the 10 to 20 year horizon, health systems will need to provide point of access services as close to the community as possible [CAHRD paper HS CTC] and systems will need to increase equity of access.

### Information

Although most people with COPD and asthma live and die in low income settings[2], much of the research relating to these diseases focuses on high-income countries. International research programmes, particularly the BOLD and ISAAC initiatives have made important steps towards addressing this knowledge gap, but some areas, notably sub Saharan Africa, have been historically under-represented[3,4].

Bronchiectasis could be considered a neglected disease from both a service and a research perspective. There are few data about the burden of disease in LMICs and limited evidence to guide practice.

The lack of academic capacity and funding for research can make building the evidence base around the epidemiology and management of CRDs most challenging.

### Human resources

Healthcare workers with appropriate knowledge and skills are needed to deliver care to people with chronic respiratory symptoms. This knowledge and skills will need to cover the clinical assessment of patients, the conduct and interpretation of investigations and the institution of a management plan. Self-management approaches are important for the management of many of these long-term conditions their degree of control is variable over time. The ability to communicate effectively with patients (overcoming educational, social and linguistic barriers) about their condition and treatment is another important skill set in this context where partnership between clients and providers is often central to success. Many patients will need support managing the identity transition to being a person with a chronic illness or with a controlled long-term health condition that requires regular treatment[19].

The ability of individuals to competently undertake specific roles is more important than job position; some of these roles could be undertaken by individuals without formal qualifications who are closer to the communities [CAHRD paper HS CTC].

Offering treatment and support close to communities is important to reduce the direct and indirect costs of disease management to the individual and the household. CTC providers also offer advantages through their social proximity in supporting individuals with the social dimensions of chronic illness [CAHRD paper HS Capacity].

### Medical products

Effective care of chronic respiratory diseases requires access to equipment and consumables needed to make and monitor diagnoses - particularly sputum examination and culture, assessment of airway physiology using peak flow meters and spirometers, chest X-rays and HRCT. Access to sputum examination and culture is especially important in the diagnostic evaluation of a patient with chronic cough and in the evaluation of patients with bronchiectasis and frequent infective exacerbations in whom less commonly encountered micro-organisms like pseudomonas can drive the process. Spirometry is essential to secure a diagnosis of COPD and is useful in the evaluation of patients with asthma. HRCT imaging is most useful for making the diagnoses of bronchiectasis and emphysema.

These diagnostic and monitoring technologies however are often unavailable in LMICs and when available, they are often unaffordable, poorly-maintained and lack quality control processes[20]. Where unavailable, pragmatic approaches to diagnostic and management decision-making have to be used, but these decision can be inaccurate and less efficient, especially where long-term treatments are initiated for incorrect diagnoses.

### Service delivery: treatments

The lack of availability of basic effective treatments for airways diseases is a key factor limiting the management of these conditions. The essential treatments in themselves are relatively straightforward as are guidelines for their use[21]. Effective chronic disease management of all the conditions requires education and partnership to ensure avoidance of triggers (e.g. allergens) and disease drivers (e.g. exposure to tobacco and other biomass smoke), sputum clearance, correct use of medication, monitoring of the disease and prompt institution of action plans in the event of exacerbation and ongoing supplies of medication that often needs to continue life-long. These interventions require health promotion in its widest sense including ‘structural interventions’ to address the underlying drivers of health risks, since the poorest and most marginalized may be those least able to make choices and to avoid triggers or drivers of disease[22] [CAHRD Paper LH Biomass].

In terms of medication, all the chronic airways diseases can benefit from access to short-acting inhaled bronchodilators with the inhaled corticosteroid forming the cornerstone of asthma management. Access to oral corticosteroids, inhaled bronchodilators, oxygen and antibiotics is needed for effective treatment of exacerbations. There are important uncertainties in the detail such as the efficacy of treatments developed for smoking-related COPD in non-smokers with COPD but in terms of getting to the point of having access to basic effective treatments it is probably reasonable to largely extrapolate from existing (e.g. tobacco) literature [CAHRD Paper LH Biomass]. One could therefore conclude that from a treatment perspective, there already exist simple effective treatments and as such the development of new treatments (unless substantially more affordable) is less important than matching current and projected needs with currently available treatments in a sustainable way. The latter is arguably the key challenge for the management of airways diseases over the 10 to 20 year horizon.

### Healthcare financing

**Figure 2:**
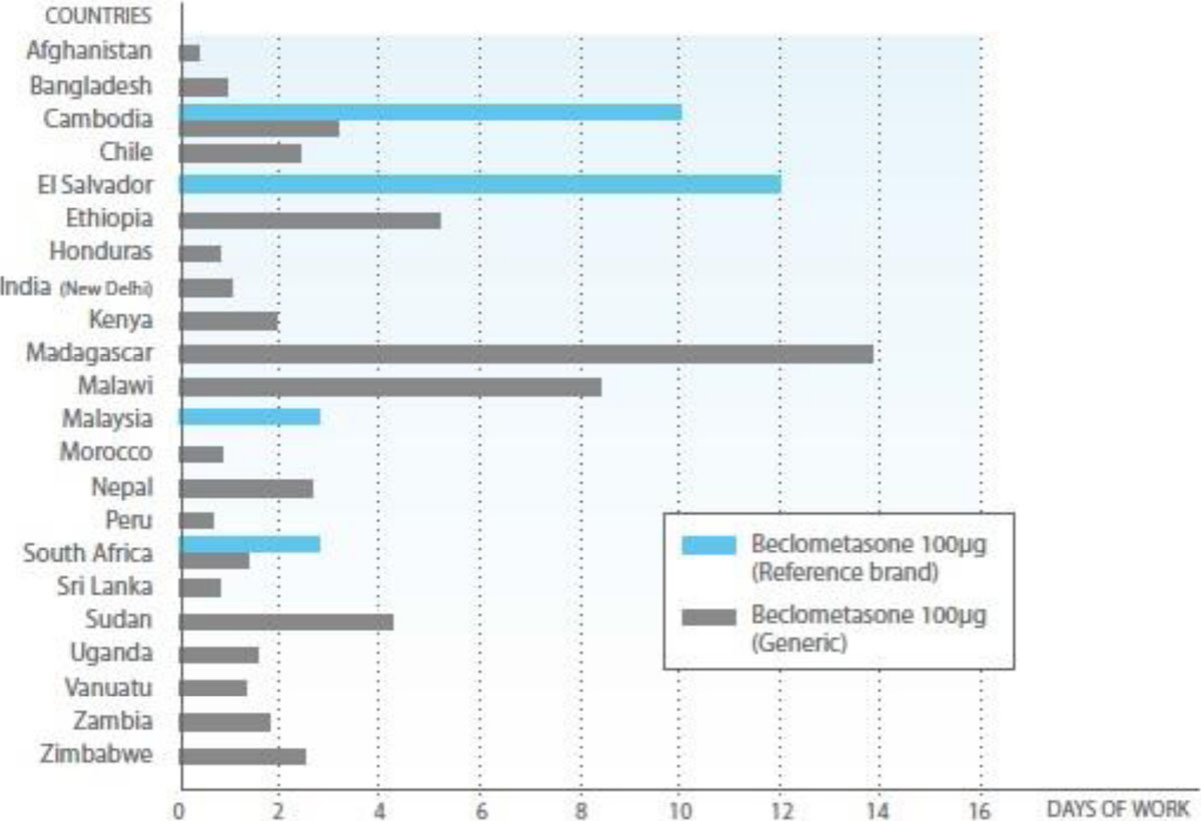
Global Asthma Report 2011 Healthcare finance graph

Affordability is a critical determinant of access to investigations and treatment for people with airways diseases. The figure below from the Global Asthma Report 2011 illustrates the affordability of a month's supply of inhaled corticosteroid for asthma purchased from a private pharmacy presented as the number of days of wages required to purchase one inhaler: Figure 2

The month on month ongoing out of pocket costs of just one inhaler can be substantial and to the point of representing a catastrophic expenditure from the household's perspective. Individuals facing such costs may find themselves stuck between such high costs for an effective treatment on the one hand vs continuing to suffer ongoing chronic symptoms and a risk of exacerbations on the other [CAHRD Paper LH Costs]. Provision for such ongoing healthcare costs needs to be made explicitly in the design of health financing modalities such as social health insurance policies and essential healthcare packages, to ensure equity in universal healthcare coverage. Careful consideration of the priorities for getting maximum benefit for the least cost for the poorest people with chronic respiratory symptoms will also be needed

### Leadership/Governance

The development of a strategic policy framework is required to enable the systematic strengthening of health systems to deliver improved care for chronic respiratory illnesses. The development of concrete chronic disease policies and oversight has been identified as an important element of developing effective chronic disease interventions[6]. The involvement of civil society, for example in the form of patient advocacy and support groups is likely to significantly shape the effectiveness of interventions and equity in outcomes[6].

## Towards solutions: the 10 to 20 year horizon

A challenge for CAHRD over the 10 to 20 year horizon is to deliver on its mission to transform health systems to improve the health of LMIC populations. The vision of a healthy future for low and middle-income populations should be realized in this timeframe.

In contrast to other neglected conditions, such as the Neglected Tropical Diseases [CAHRD Papers NTD Tools, Models, Delivery], basic diagnostic tools and treatments for chronic respiratory diseases have been available for decades. The immediate priority therefore is to match current diagnostics and treatments with the people most in need of them. This will require a step change in the implementation of health systems that overcome the obstacles to access diagnosis and treatment and minimize the social and economic costs of accessing care.

There are many health system approaches that could be shared to manage chronic non-communicable (especially diabetes, heart disease and stroke), infective (especially HIV) and chronic respiratory diseases. There is thus a large opportunity to develop integrated approaches for the efficient management of chronic respiratory and non-communicable chronic diseases together with more traditional vertical programs such as TB, malaria and HIV. Such integrated approaches would build on the 2015 development goals and would be consistent with the vision of Universal Health Care

A health system responsive to the needs of patients with chronic diseases will require provision for the management of acute situations, when the control of the underlying condition is lost. Acute exacerbations are common with airways diseases; patients with diabetes experience hypo and hyperglycaemic emergencies and patients with ischaemic heart disease and cerebrovascular disease can suffer myocardial infarction and strokes. Similar parallels can be drawn with the management of HIV, with life-long treatment punctuated by the emergence of acute complications such as opportunistic infections and drug resistance.

Health services management of these chronic diseases will need to be located within a multi-faceted and multi-institutional framework, enabling intervention and collaboration across structural, community and individual levels; for example between policy makers, industry, the media, community organisations such as patient advocacy and support groups, and individual patients[6].

To reach the objective of developing effective and affordable health systems responsive to the management of chronic respiratory diseases will require informed policy and decision-making. However the evidence base to generate these policies and to inform the structures of a health system that are needed for their management is weak. These weaknesses herein constitute a strategic opportunity for CAHRD to conduct health systems research to develop innovative approaches and inform the processes. For this to be a success there will be a need to strong South-North and South-South collaboration and capacity strengthening for multi-disciplinary research and service delivery.

All of these activities will need to take place in a changing world where increasingly health issues are shared and converging. There has never been a greater opportunity to improve the health and well-being of people living in LMICs for the benefit of all as part of what has been coined the Grand Convergence between populations across the world.

## Research opportunities

There is a need for epidemiological studies to determine the community burden of chronic respiratory diseases in LMICs and to understand the underlying causes of these conditions. CAHRD investigators (through LSTM and MLW collaborations and a public-private partnership with GlaxoSmithKline) are currently conducting rural and urban BOLD studies in Malawi. These include studies to understand the impact of household air pollution on the decline in lung function over time. However more studies, including populations with different epidemiological characteristics are needed. Burden of disease studies should include sub-studies on the social and economic costs of disease and treatment to guide resource allocation. Studies of chronic respiratory diseases could be integrated within studies for other chronic diseases; and integrated approaches to improving population health should be encouraged.

Health systems research is needed to generate solutions to obstacles to access health care. These studies should focus on the poorest and most vulnerable populations of LMIC who are often less likely to benefit by health system improvements driven by more affluent sectors of society. Herein lies the importance of exploring the effectiveness and cost effectiveness of close-to-community interventions that reach out to these groups. CAHRD has a strong presence in this area. For example, the *Triage* study in Malawi and Sudan focused on CTC involvement in TB and HIV referral and follow on Triage Plus studies, which focus on CTCs and respiratory diseases, are underway. Studies in Ethiopia have demonstrated that CTC approaches can significantly increase and improve access to TB diagnosis and treatment and have been declared trail finder approaches by the WHO and the Health Assembly [CAHRD paper HS CTC].

Studies that facilitate understanding the barriers to accessing care, and achieving long-term management of a variety of chronic health conditions and social groups, are needed to ensure that services are appropriately designed, acceptable and tailored to the needs of stakeholders. Such studies will need to include explorations of the ‘social logic’ of illness conceptualization and how individuals act to manage their conditions within specific social contexts[19]. Qualitative studies and eHealth studies should facilitate the design of effective communication around long-term disease and exacerbation management and outcome and process evaluations of complex health systems interventions are needed.

## Conclusions

Chronic respiratory diseases are major global health problems causing substantial morbidity and mortality and their burden will increase over the 10 to 20 year horizon. Many individual, societal and economic obstacles prevent poor populations with chronic respiratory symptoms accessing effective health care. Health systems are inadequately resourced and developed to respond to the needs of these conditions. Solutions to address these obstacles are feasible but require the development of an evidence base to inform effective and implementable policy and decision-making. Research and health system capacity strengthening are essential building blocks to achieving the vision of improving the health of LMICs.

## Competing interests

The authors declare that they have no competing interests.

## Authors' contributions

KM led on writing of the paper; all authors contributed to the development of the final manuscript.

## Declarations

This article has been published as part of *BMC Proceedings* Volume 9 Supplement 10, 2015: The 2014 CAHRD (Collaboration for Applied Health Research & Delivery) Consultation. The full contents of the supplement are available online at http://www.biomedcentral.com/bmcproc/supplements/9/S10.
